# The effect of risperidone on reward‐related brain activity is robust to drug‐induced vascular changes

**DOI:** 10.1002/hbm.25400

**Published:** 2021-03-05

**Authors:** Peter C. T. Hawkins, Fernando O. Zelaya, Owen O'Daly, Stefan Holiga, Juergen Dukart, Daniel Umbricht, Mitul A. Mehta

**Affiliations:** ^1^ Department of Neuroimaging Institute of Psychiatry, Psychology and Neuroscience, King's College London London UK; ^2^ Roche Pharma Research and Early Development Roche Innovation Centre Basel, F. Hoffmann‐La Roche Ltd. Basel Switzerland; ^3^ Institute of Systems Neuroscience, Medical Faculty, Heinrich Heine University Düsseldorf Düsseldorf Germany

**Keywords:** antipsychotic, ASL, breath‐hold, cerebrovascular, dopamine, fMRI, MID, reward

## Abstract

Dopamine (DA) mediated brain activity is intimately linked to reward‐driven cerebral responses, while aberrant reward processing has been implicated in several psychiatric disorders. fMRI has been a valuable tool in understanding the mechanism by which DA modulators alter reward‐driven responses and how they may exert their therapeutic effect. However, the potential effects of a pharmacological compound on aspects of neurovascular coupling may cloud the interpretability of the BOLD contrast. Here, we assess the effects of risperidone on reward driven BOLD signals produced by reward anticipation and outcome, while attempting to control for potential drug effects on regional cerebral blood flow (CBF) and cerebrovascular reactivity (CVR). Healthy male volunteers (*n* = 21) each received a single oral dose of either 0.5 mg, 2 mg of risperidone or placebo in a double‐blind, placebo‐controlled, randomised, three‐period cross‐over study design. Participants underwent fMRI scanning while performing the widely used Monetary Incentive Delay (MID) task to assess drug impact on reward function. Measures of CBF (Arterial Spin Labelling) and breath‐hold challenge induced BOLD signal changes (as a proxy for CVR) were also acquired and included as covariates. Risperidone produced divergent, dose‐dependent effects on separate phases of reward processing, even after controlling for potential nonneuronal influences on the BOLD signal. These data suggest the D2 antagonist risperidone has a wide‐ranging influence on DA‐mediated reward function independent of nonneuronal factors. We also illustrate that assessment of potential vascular confounds on the BOLD signal may be advantageous when investigating CNS drug action and advocate for the inclusion of these additional measures into future study designs.

## Graphical abstract

Single dose risperidone produces dose‐response and divergent effects on different phases of reward‐related brain activity in healthy human volunteers in a placebo‐controlled fMRI study. These effects persist after accounting for potential nonneuronal effects of the drug on cerebral blood flow and cerebrovascular reactivity (as measured by a breath hold challenge).

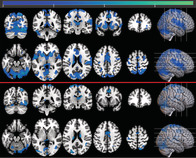

## 1 INTRODUCTION

The cortical‐striatal circuits modulated by the neurotransmitter dopamine (DA) have been consistently linked with several critical facets of human function. DA neurons project from the midbrain DA nuclei, innervating subcortical and cortical systems, and form a central component of the human reward system (Haber & Knutson, 2010). Neuroimaging tools have expanded our knowledge of the structural and functional characteristics of these systems in vivo, and how they may be altered in individuals with psychiatric disorders (Peters, Dunlop, & Downar, 2016; Radua, Schmidt, Borgwardt, & et al., 2015). These systems are also sensitive to pharmacological challenges, which is a useful tool for scrutinising the modulation of reward‐related activity when combined with techniques such as functional Magnetic Resonance Imaging (fMRI [Ogawa, Lee, Kay, & Tank, 1990]). Several studies in healthy human volunteers have reported alterations in the reward‐related Blood Oxygen Level Dependent (BOLD) signal in both subcortical and cortical regions following the introduction of compounds which manipulate DA signalling, such as the dopamine D2 receptor antagonists amisulpride (Admon et al., 2017), haloperidol (Pessiglione, Seymour, Flandin, Dolan, & Frith, 2006), olanzapine (Abler, Erk, & Walter, 2007), lurasidone (Wolke et al., 2019) and sulpiride (Diederen et al., 2017); the indirect DA agonists dextroamphetamine (Knutson et al., 2004) and amphetamine (O'Daly et al., 2014); and the DA precursor L‐DOPA (Pessiglione et al., 2006). Antipsychotic medications are a prime example here, as their interaction with the DA system (particularly in reward and salience brain networks) is thought to be important for both the therapeutic and side effect profile of these interventions. For instance, changes in the reward anticipatory BOLD signal produced by DA modulators during the Monetary Incentive Delay (MID) task (Knutson, Westdorp, Kaiser, & Hommer, 2000) have been correlated with symptom change in schizophrenia (Juckel, Schlagenhauf, Koslowski, Filonov, et al., 2006a; Nielsen et al., 2012; Walter, Kammerer, Frasch, Spitzer, & Abler, 2009) demonstrating the potential clinical relevance of being able to determine drug‐induced changes in brain activity.

As several reports have suggested (Iannetti & Wise, 2007; Lu, Yezhuvath, & Xiao, 2010; Lu, Zhao, Ge, & Lewis‐Amezcua, 2008) interpretation of drug‐induced changes in the BOLD signal can be confounded by additional, nonneuronal factors. Drug effects on BOLD contrast may be obfuscated by the drug influencing one or more of the elements within the neurovascular cascade that putatively links the signal with the underlying neural activity. For instance, an undetected drug‐induced modulation of baseline cerebral blood flow (CBF), vascular signalling or cerebrovascular reactivity (CVR; the ability of cerebral vasculature to modulate blood flow in response to vasoactive stimuli) could produce a BOLD response in the absence of a change in neural activity, or “mask” an actual neuronal response. This issue is particularly relevant when examining DA modulators, as single dose D2 antagonists have been repeatedly shown to produce rapid alterations in cerebral blood flow in humans (Fernández‐Seara et al., 2011; Handley et al., 2013; Hawkins et al., 2018; Mehta et al., 2003).

There has been a paucity of experimental studies examining the direct impact that these haemodynamic factors have on the interpretability of BOLD changes induced by DA modulators using task‐based fMRI. Methylphenidate (a DA reuptake inhibitor) did not alter motor cortex BOLD activation during a finger tapping task, nor did it alter CBF in the same region (Rao et al., 2000). Single dose olanzapine in healthy volunteers was found to alter breath‐hold induced BOLD signal changes (a proxy of CVR) in cortical areas, but these changes were not apparent in those areas where the drug altered reward task elicited BOLD signal (Abler et al., 2007). Neither study included the CBF/breath‐hold data directly in the analysis of the task activated BOLD. Several recommendations have been made in recent years to address the potential impact that pharmacological compounds may have on neurovascular coupling (Bourke & Wall, 2015; Iannetti & Wise, 2007; Jenkins, 2012)—accounting for baseline CBF, assessing vascular reactivity and including a placebo condition have all been proposed as minimum recommendations for pharmacological MRI—however, it remains rare that these factors are addressed together.

Furthermore, the majority of imaging studies examining the effect of antipsychotics on reward function are conducted in often highly heterogenous clinical cohorts with a known baseline disruption to dopaminergic function, which may further cloud interpretation. Observing the modulatory effect dopaminergic medication has in healthy humans removes this confound, in addition to affording a higher level of experimental control by more readily allowing placebo control. Therefore, in order to clarify the effect of clinically prescribed DA drugs on brain reward function, this study aimed to examine the effect of a commonly prescribed antipsychotic on the BOLD response to the MID task in healthy volunteers, while attempting to assess and account for likely nonneuronal drug effects on the BOLD signal. We extended the approach taken by Abler et al., (2007) and employed an experimental, placebo‐controlled design in healthy humans, using a single dose of the D2 antagonist risperidone. Each participant was given two doses of this drug on separate sessions—a clinically relevant dose of 2 mg and a smaller dose of 0.5 mg—in addition to a placebo session, in order to assess any dose‐dependent effects. Each participant was scanned while performing the extensively used MID task, which has proven sensitivity to DA system activation and manipulation (Bjork, Grant, Chen, & Hommer, 2014; D'Ardenne, McClure, Nystrom, & Cohen, 2008; Knutson & Gibbs, 2007; Schott et al., 2008; Ye, Hammer, Camara, & Munte, 2011). We also collected whole brain, high‐resolution regional cerebral perfusion maps (using ASL) and estimated cerebrovascular reactivity (using a breath‐hold task). Drug induced changes in reward‐related BOLD contrast were assessed both with and without the inclusion of these covariates at voxelwise and ROI level. Based on an earlier study (Abler et al., 2007), we hypothesised that antipsychotic administration would reduce BOLD activation during reward anticipation during the MID task in striatal regions. Effects on reward outcome processing were also explored, as DA manipulation of this phase of the task has been less frequently reported.

## 2 METHODS

### 2.1 Participants

21 healthy right‐handed male participants (age range 19–41, mean age 27.56 ± 6.87 years) were recruited using newspaper/radio advertisements. Screening procedures were conducted between 28 and 2 days before the first imaging session and assessed general suitability for the study (see Data S1). The study was approved by the London (Brent) Human Research Ethics Committee (REC reference: 13/LO/1183).

Participants who met eligibility criteria were scanned three times in total. During each visit, participants received either a single oral dose of risperidone 0.5 mg, risperidone 2 mg or placebo 2 hr prior to their scan, with the scan taking place at the estimated peak plasma concentration of the drug (de Greef, Maloney, Olsson‐Gisleskog, Schoemaker, & Panagides, 2011). Two milligram achieves D2 receptor occupancy in the clinically effective range (~60% [Kapur, Zipursky, Jones, Remington, & Houle, 2000]). A minimum of 7 days separated each scan to allow wash‐out of each compound. Scans were conducted at the same time of day at each visit. Within‐group treatment order was randomised using a Williams square design. As a measure of subjective sedation, participants rated their own alertness using a Visual Analogue Scale (VAS; Herbert, Johns, & Dore, 1976).

### 2.2 Image acquisition and preprocessing

Full details of acquisition, preprocessing, modelling and analysis of all imaging data can be found in Data S1.

All scans were conducted on a GE MR750 3‐Tesla scanner using a 12‐channel receive‐only head coil. Functional scans (MID and breath‐hold) were carried out using a temporal series of Gradient‐Recalled Echo Planar Imaging (GE‐EPI) whole brain scans, each comprising of 38 near‐axial slices, with an isotropic spatial resolution of 3.3 mm (TR = 2000 ms; TE = 28 ms; flip angle = 75°; number of volumes = 414 [MID], 146 [breath‐hold]; FoV = 214 mm).

Preprocessing was conducted in the Statistical Parametric Mapping (SPM) analysis suite, issue 12, on Matlab 8.2.0.701, and included resetting of image origins, slice time correction, two‐pass realignment, co‐registration and normalisation to MNI space using DARTEL (Diffeomorphic anatomical registration through exponentiated lie algebra [Ashburner, 2007]), and smoothing using an 8 mm FWHM kernel (see Data S1 for full details).

The breath‐hold task provided an estimate of CVR and was administered and modelled as described previously (Birn, Smith, Jones, & Bandettini, 2008; Murphy, Harris, & Wise, 2011; Thomason & Glover, 2008; Urback, MacIntosh, & Goldstein, 2017). Participants were instructed to follow a simple set of instructions on screen alternating between paced breathing (45 s) and breath holding (16 s), with this cycle repeated five times (data from the first cycle was discarded to eliminate nonsteady state effects of the paradigm on the BOLD signal). The breath hold challenge was modelled with box‐car function regressors for paced and held breathing, but incorporated a delayed onset of 9 s and included the temporal derivatives, as previously shown to provide the most accurate modelling of vascular reactivity (Murphy, Harris, & Wise, 2011). Whole brain maps of the contrast held > paced breathing provided a metric of CVR.

Whole brain maps of regional CBF were obtained using an ASL methodology previously reported (Hawkins et al., 2018). Full details on acquisition, modelling, analysis and monitoring of participant adherence of the ASL and breath‐hold data is available in the Data S1.

The MID has extensively used to elicit and study reward‐related activation within fMRI designs (Knutson & Greer, 2008), and has been shown to be reliable over time in healthy volunteers (Plichta et al., 2012). The version used in this study is most closely comparable to that used in (Knutson, Fong, Adams, Varner, & Hommer, 2001) and was modelled as outlined in Abler et al. (2007), with full details in the Data S1. Weighted contrasts of interest were set to explore main effect of anticipation of reward (High Cue & Low Cue > Neutral Cue [0.5 0.5 > −1]), and main effect of receipt of reward (High Win & Low Win > High no‐win & Low no‐win [0.5 0.5 > −0.5 −0.5]).

Recent evidence suggests traditional parametric statistics in whole brain analysis may be at risk of inflating the false positive rate (Eklund, Nichols, & Knutsson, 2016). To address this, we employed nonparametric permutation testing to explore whole‐brain drug effects, which does not rely on any assumptions of normality. Nonparametric voxelwise analysis of drug effect on reward elicited BOLD involved paired sample *t*‐tests of high dose versus placebo, and low dose versus placebo on the MID task using the *RANDOMISE* feature in FSL with threshold free cluster enhancement (TFCE [Smith & Nichols, 2009]), both with and without inclusion of voxelwise covariates of CBF and the breath‐hold task as a measure of CVR. This method allows the calculation of a unique GLM at each voxel which includes the session relevant covariates from the CBF and breath‐hold maps as they are DARTEL normalised to the same MNI resolution as the MID contrast maps. Dose versus placebo *t* tests were chosen in favour of a full ANOVA model due to the repeated measure nature of the data and the assumptions of compound symmetry made by the analysis software. We therefore complemented this analysis with a linear mixed model analysis of carefully selected a priori reward system ROIs.

Five reward system related bilateral ROIs were defined for analysis of reward anticipation (ventral striatum (VS), caudate, putamen, ventral tegmental area (VTA) and amygdala) with an additional two ROIs added to these for the assessment of reward outcome activity (Ventromedial prefrontal cortex vmPFC) and anterior cingulate cortex (ACC)). Full details of the definition of these ROIs is available in the Data S1.

The mean beta estimates created from the MID first‐level modelling described above (anticipation of reward [win cue vs. neutral cue] and receipt of reward [win outcome vs. no win outcome]) were extracted from within each of the ROIs using the MarsBar plugin in SPM12, and were used to assess drug induced changes in MID activity for each drug and placebo session. The same ROI data was extracted from each of the CVR and breath‐hold maps. Extracted values from these ROIs were analysed with a linear mixed effects model using the *lme4* package in R (version 3.6.3; February 29, 2020), with Dose and ROI added as fixed factors and Subject as random factor. A second model which included the extracted CBF and CVR metrics as covariate fixed factors was also conducted. The *emmeans* package in *R* was used to calculate estimated marginal means from the model, and post‐hoc pairwise comparisons were conducted between each of the dose levels across ROIs (using the pairs function within *emmeans*) to assess differences between each drug or placebo session, with Tukey's method for comparing a family of three estimates used for *p* value adjustment. Due to the way variance is partitioned in linear mixed models, obtaining precise variance estimates for individual model terms is not straightforward—however, to give an indication of the contribution of the predictors in the models with and without the vascular covariates included, a conditional *R* ^2^ for each model was calculated using the MuMIn R package as described in Nakagawa and Schielzeth (2013).

## 3 RESULTS

Four participants were removed from group analysis due to not performing the MID task adequately during one or more sessions according to our a priori threshold, leaving 17 participants in the analysis.

### 3.1 Effect of drug on alertness and task performance

A one‐way repeated measures ANOVA of the alertness subscale of the VAS revealed no significant effect of risperidone (*F*[2,32] = 2.458, *p* = .102). There was no significant effect of drug on task performance (*F*[2,32] = 2.048, *p* = .146), as measured by hit rate (percentage of monetised trials successfully won).

### 3.2 Effect of risperidone on breath‐hold task

**FIGURE 1:**
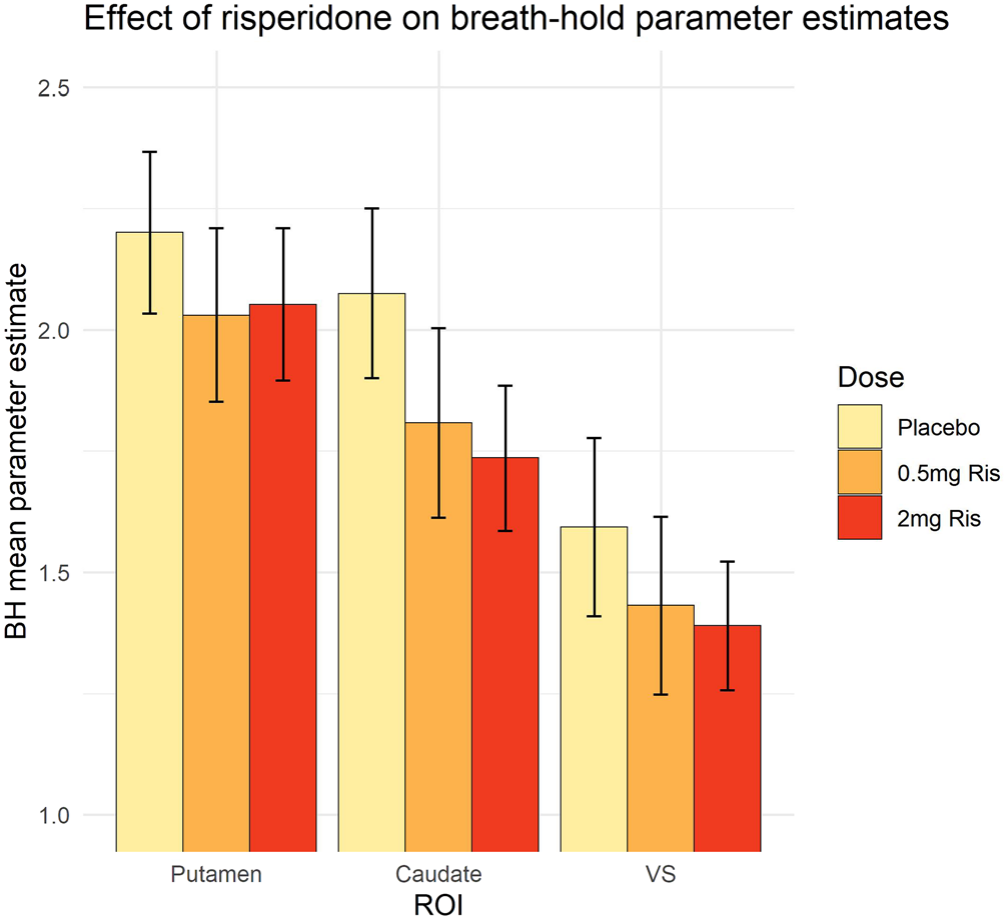
Effect of risperidone on breath‐hold parameter estimates (with SE bars) across striatal ROIs (*n* = 17)

Participants adhered to the breath‐hold task well and the timing and extent of breath‐holding was consistent across drug sessions (see Data S1 & Figure S3). The task elicited widespread significant increases in BOLD signal throughout the grey matter during periods of breath‐hold (see Figure S4), but the whole brain analysis of the effect of risperidone did not reveal a significant effect of drug on the BOLD changes produced by the task. Extracted parameter estimates of each ROI from contrast maps of the breath‐hold task (as a proxy for CVR) were analysed with a linear mixed effect model, with ROI as fixed effect, dose as fixed effect and subject as random effect. In this initial model there was no main effect of dose or dose*ROI interaction. However, the same model focusing on just the three striatal ROIs (the primary regions implicated in MID task BOLD changes) revealed a main effect of dose (*F*[2,128] = 4.017, *p* = .020). All three striatal ROIs had a reduction in CVR parameter estimates after risperidone exposure (Figure 1). Pairwise comparisons conducted between dose levels on CVR averaged across the striatal ROIs revealed the only significant difference was following 2 mg risperidone compared to placebo (reduction of 0.0271, *p* = .027) after correction for multiple comparisons between the three dose levels. Further pairwise comparisons within each ROI revealed 2 mg risperidone produced the largest reduction compared to placebo in the caudate, although this did not survive correction for multiple comparisons between the three dose levels (a reduction of 0.34, *p* = .07).

### 3.3 Effect of risperidone on regional cerebral perfusion (CBF)

As previously reported (Hawkins et al., 2018), 2 mg risperidone produced significant increases in striatal blood flow, with a large continuous cluster centred around the left caudate extending into bilateral caudate, putamen and anterior cingulate. The 0.5 mg risperidone dose produced a similar but less pronounced pattern to that seen after the 2 mg dose and was limited to left and right caudate and putamen (see Figures S5 & S6).

**FIGURE 2:**
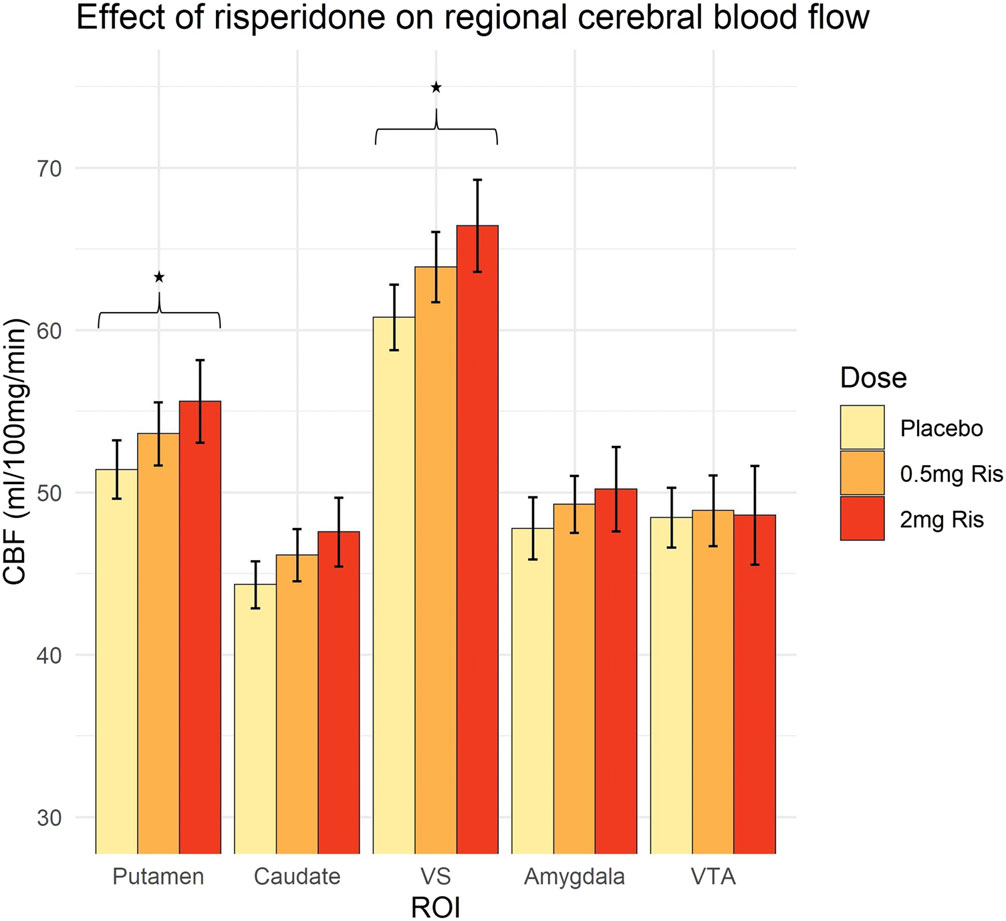
Effect of risperidone on CBF (with SE bars) across ROIs (*n* = 17). *Pairwise comparison significant *p* < .05 after multiple comparison correction between dose levels within each ROI

Extracted CBF values from the reward anticipation ROIs were analysed as above and revealed a main effect of dose (*F*[2,224] = 13.051, *p* < .001) and no interaction between ROI*Dose. Planned pairwise comparisons conducted between each of the three dose levels on CBF averaged across the ROIs revealed the largest significant difference was following 2 mg risperidone compared to placebo (increase of 3.13 ml/100 mg/min, *p* < .0001), followed by 0.5 mg risperidone compared to placebo (increase of 1.81 ml/100 mg/min, *p* = .009) after correction for multiple comparisons between the three dose levels. Pairwise comparisons within each ROI revealed the largest increases in CBF following 2 mg risperidone compared to placebo were localised to the striatal ROIs (Figure 2), specifically the putamen (increase of 4.20 ml/100 mg/min, *p* = .007), ventral striatum (increase of 5.65 ml/100 mg/min, *p* < .0001) and caudate (increase of 3.25 ml/100 mg/min, *p* = .0501) after correction for multiple comparisons between the three dose levels (see Figure 2).

### 3.4 Effect of risperidone on reward‐related fMRI

#### 3.4.1 Reward anticipation

**FIGURE 3:**
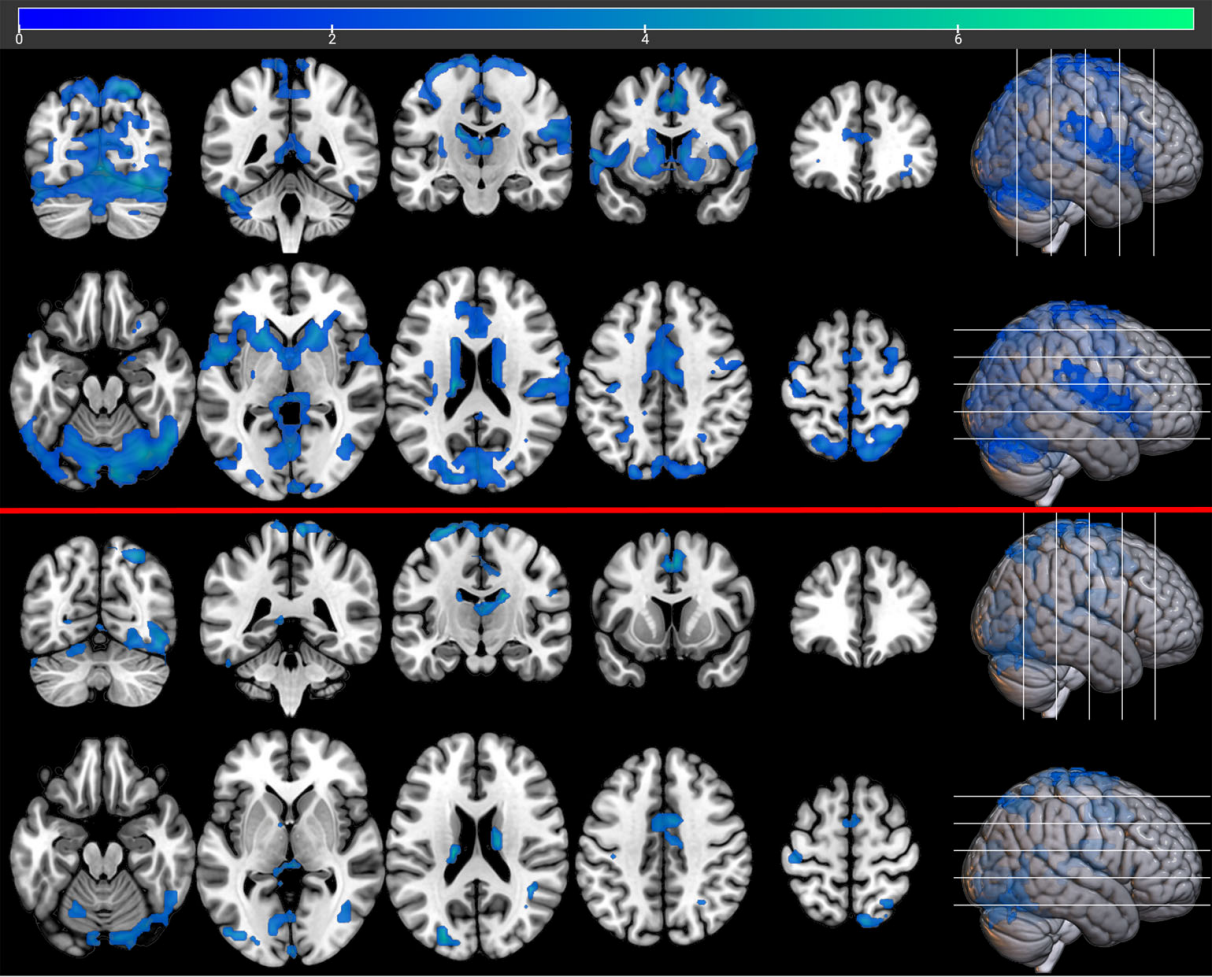
Effect of Placebo >2 mg Risperidone on Reward anticipation (*n* = 17), before (top) and after inclusion of voxelwise vascular covariates (Whole brain permutation testing, FWE corrected *p* < .05, 5,000 permutations). Colour bar denotes voxelwise paired sample *t* statistic. Top: Placebo > Risperidone 2 mg *|* Bottom: Placebo > Risperidone 2 mg with voxelwise CBF and CVR maps included as covariates

Nonparametric whole brain analysis revealed a significant effect of 2 mg risperidone on reward anticipation compared to placebo, reducing activation in the caudate, putamen, ventral striatum cingulate and thalamus, in addition to visual and supplementary motor cortex (top panel, Figure 3). 0.5 mg risperidone did not produce any significant changes in BOLD contrast although reducing the threshold to a lower than recommended exploratory level of *p* = .1 (FWE corrected) revealed a similar spatial pattern of changes to those produced by 2 mg risperidone.

The extracted reward anticipation parameter estimates from the a priori ROIs were analysed as above, which revealed a significant main effect of dose (*F*[2,224] = 15.898, *p* < .001) and no interaction between ROI*Dose. Planned pairwise comparisons were conducted between each of the three dose levels on parameter estimates averaged across the level of ROI and revealed the largest significant difference was following 2 mg risperidone compared to placebo (decrease of 0.224, *p* < .0001), while there were also significant differences following 0.5 mg risperidone compared to placebo (decrease of 0.116, *p* = .011) and following 2 mg compared to 0.5 mg (decrease of 0.108, *p* = .02), after correction for multiple comparisons between each of the three dose levels. Comparisons within each ROI revealed the largest decreases in reward anticipation following 2 mg risperidone compared to placebo were localised to the striatal ROIs, specifically the putamen (decrease of 0.2176, *p* = .040), ventral striatum (decrease of 0.2356, *p* = .023) and caudate (decrease of 0.4220, *p* < .001) after correction for multiple comparisons between doses within each ROI.

The whole brain permutation testing was repeated with the addition of the voxelwise covariate maps for CBF and CVR (bottom panel, Figure 3) which resulted in a less widespread pattern of reduction in activity due to risperidone, with the striatal changes now mostly absent—although as this voxelwise analysis does not permit an interaction term these results constitute a nonquantitative representation of the magnitude of the reduction in the spatial extent of those changes.

**FIGURE 4:**
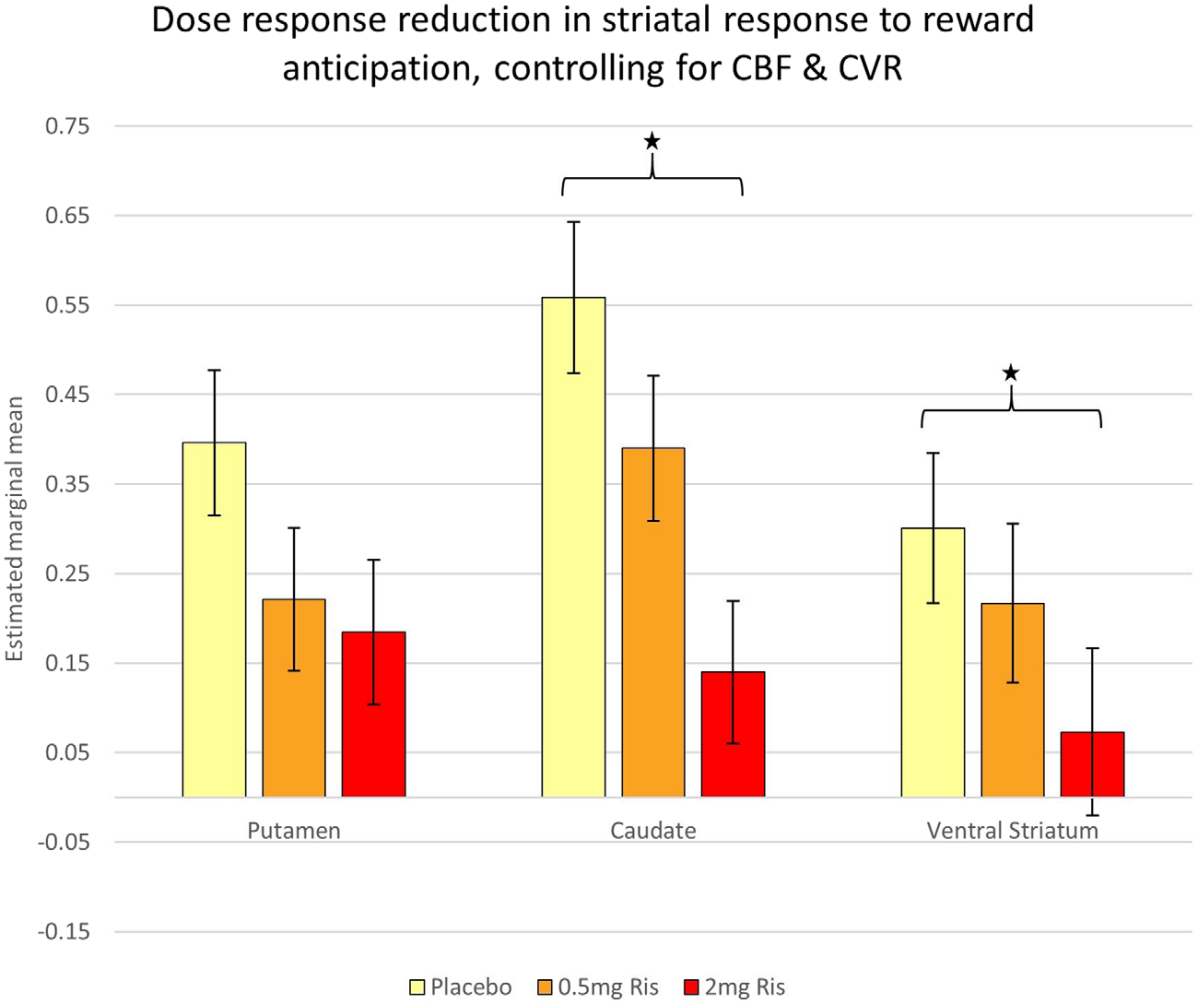
Marginal means (and SE bars) of reward anticipation beta estimates from model including CBF and CVR covariates. *Pairwise comparison significant *p* < .05 after multiple comparison correction between dose levels within each ROI

The linear mixed effects ROI model was repeated, with CBF and CVR added as fixed effects along with ROI and Dose, and Subject as random effect. The main effect of Dose remained (*F*[2,227] = 14.022, *p* < .0001) and there was no ROI*dose interaction. There was no significant main effect of CBF or CVR on the MID parameter estimates in this model overall, indicating changes in CBF or CVR alone did not significantly alter the MID parameter estimates. Planned pairwise comparisons between each of the three sessions on parameter estimates averaged across the level of ROI indicated a similar pattern as the model without the vascular covariates: the largest significant difference was following 2 mg risperidone compared to placebo (decrease of 0.219, *p* < .0001), while there were also significant differences following 0.5 mg risperidone compared to placebo (decrease of 0.113, *p* = .015) and following 2 mg compared to 0.5 mg (decrease of 0.106, *p* = .023), after correction for multiple comparisons between each of the three dose levels. Further comparisons within the ROIs indicated the largest decreases in reward anticipation following 2 mg risperidone compared to placebo were still within the striatal ROIs of the caudate (decrease of 0.4186, *p* < .001), ventral striatum (decrease of 0.2356, *p* = .036) and putamen (decrease of 0.2119, *p* = .052), with the putamen no longer significant after correction for multiple comparisons between doses within each ROI (Figure 4).

In order to gauge the contribution of the vascular covariates to the ROI models, an estimate of conditional *R* ^2^ (accounting for the fixed and random effects) was calculated for each model. The model without the covariates had a *R* ^2^ of 0.4507 while the model including the CVR and CBF covariates was 0.4533, indicating the extra variance in MID BOLD explained by the inclusion of the vascular covariates was minimal.

#### 3.4.2 Reward outcome

**FIGURE 5:**
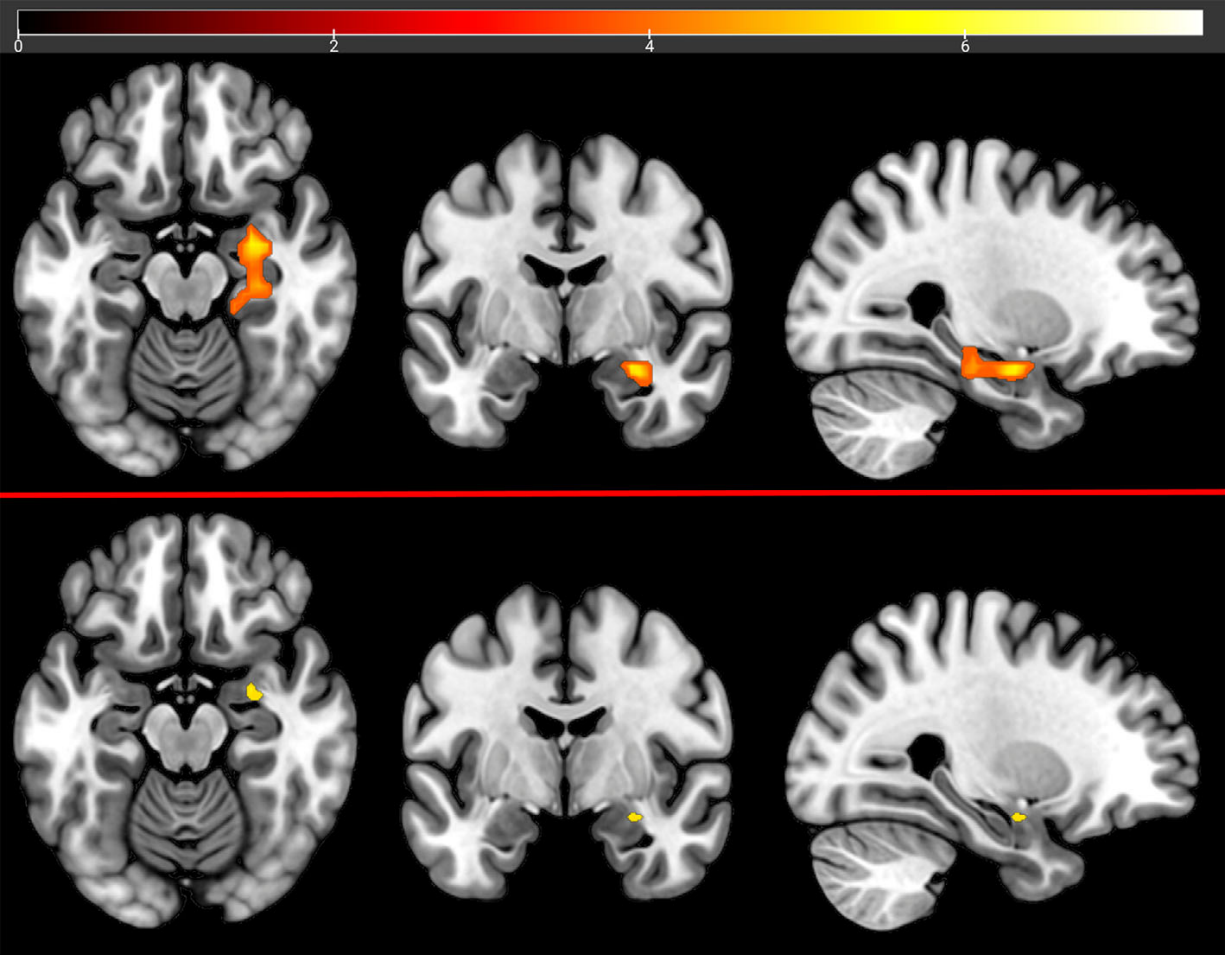
Effect of 2 mg Risperidone > Placebo on Reward Receipt (*n* = 17), before (top) and after correction for vascular covariates (Whole brain permutation testing, FWE corrected *p* < .05, 5,000 permutations). Colour bar denotes voxelwise paired sample *t* statistic. Top: Risperidone 2 mg > Placebo | Bottom: Risperidone 2 mg > Placebo with voxelwise CBF and CVR maps included as covariates

During the outcome phase, 2 mg risperidone produced a divergent effect in BOLD contrast to that seen during reward anticipation, with an *increase* in activation centred around the anterior hippocampus and amygdala (top panel, Figure 5). 0.5 mg risperidone did not produce any significant changes. Following inclusion of the vascular covariates, this was reduced to a smaller cluster of voxels centred around the amygdala (bottom panel, Figure 5). There were no significant clusters for 0.5 mg risperidone. ROI analysis did not reveal any significant results in the a priori regions during reward outcome, either before or after addition of vascular covariates.

## 4 DISCUSSION

Here we report an acute, dose‐dependent cerebral response to a commonly prescribed antipsychotic on reward processing, which appears to differentially influence the BOLD signal response during separate stages of the MID task. Risperidone produced dose–response alterations to CBF as previously reported (Hawkins et al., 2018), and breath‐hold BOLD (as a proxy for CVR) was altered by the drug in striatal areas. Both whole brain and ROI analyses revealed single dose risperidone resulted in dose‐related reductions in activation during reward anticipation in multiple reward‐relevant brain regions, while there were increases in activation during reward outcome localised to the amygdala. Accounting for CBF and CVR influenced the results in these areas, but importantly many of these changes were still detectable at both whole brain and ROI level. Using a noninvasive design, this study indicates that the potential direct and indirect effects of these drugs on the vasculature itself are measurable and can be accounted for in second level analysis.

### 4.1 Effect of risperidone on cerebral blood flow and cerebrovascular reactivity

Although the ROIs explored here suggest the influence of the vascular covariates appear to be limited, it must also be considered that not all antipsychotics may affect the BOLD response in the same way. For example, haloperidol produces a larger increase in CBF in striatal areas than that elicited by risperidone, while olanzapine does not produce changes to the same extent (Hawkins et al., 2018). This has important implications for patient studies using fMRI to assess cognitive or drug function as they will typically include cohorts taking different antipsychotics.

The direction of change in the BOLD signal in the whole brain data, observed in this work after inclusion of regional CBF as covariate (with a reduction in the amplitude and spatial extent of BOLD signal change), is consistent with what has been previously proposed regarding the effect of baseline CBF on the BOLD response. Earlier work by Simon and Buxton (2015) and Lu et al. (2008) both propose that an increase in baseline CBF (as we detected after administration of risperidone), leads to a reduction in the amplitude of the BOLD signal. This is wholly consistent with our observations, although the earlier caveat regarding the qualitative nature of the comparison of the whole brain data applies here. Alternatively, it may be that some of the drug effects are no longer strong enough to survive the correction for multiple comparison at whole brain level in some regions after the covariates are included. This could be for a number of reasons, such as a reduction of power due to the inclusion of the covariates or a reduced effect size. The ROI analysis revealed risperidone modulation of reward‐related BOLD both before and after the covariates are included, which could suggest that carefully preselected ROIs may be preferable in these studies.

The hypercapnia produced by the breath‐hold task is assumed to induce an increase in CBF without affecting CMRO2, as the increase in carbon dioxide in the blood produces vasodilation. The related BOLD changes in response to hypercapnia (Figure S4) are therefore primarily reflective of an increase in CBF in the absence of any meaningful neuronal activity or oxygen metabolism (relative to the task condition). This hypercapnic response is often used as a method to attempt to correct for inter‐subject variability in the BOLD signal, whereby dividing the functional BOLD response by the hypercapnic BOLD response or using the hypercapnic BOLD response as a covariate gives a normalised BOLD response (Bandettini & Wong, 1997; Liau & Liu, 2009). In this study we are not attempting to normalise the BOLD response per se, but illustrate the potential effect the drug may be having on processes related to cerebrovascular reactivity (a known mediator of the BOLD signal), and attempt to account for this when examining the drug effect on reward system function. If the BOLD response to the breath‐hold induced increase in vasoactive CO_2_ is altered by the presence of a drug (as a result of drug‐mediated interference with some element of the signalling cascade between neurons and/or glial cells and the vasculature), the concern is this may also occur with vasoactive signallers that are released in response to changes in neuronal activity and which mediate neurovascular coupling. Risperidone itself did not produce a detectable voxelwise effect on CVR as measured with the breath‐hold task, although changes were observed in the striatal ROIs—covarying for this in the ROI models did not remove the effect of the drug on reward‐related BOLD. In comparison, Abler et al. (2007), reported a significant effect of olanzapine on vascular reactivity in insula, cingulate and occipital cortex ROIs, but no effect in a ventral striatum ROI. Olanzapine has an extended receptor profile compared to risperidone, which may explain the discrepancy with our findings—DA is of course not the only neurotransmitter mediated by antipsychotics, and interaction with other neurotransmitter systems such as serotonin and histamine are highly likely to be involved with the effects observed here. Examination of the effect on the BOLD response to breath‐hold of different antipsychotics with a different range of specific receptor profiles would help characterise their potential influence on the BOLD signal more clearly.

One assumption made by controlling for CBF and CVR in the fashion reported here is that a linear and independent association exists between the two, which may only be an approximation of their relationship. Liu et al. (2013) characterised the role of global venous oxygenation and CBF, as well as local CVR and resting state fluctuation amplitude, showing they accounted for 42–74% of the BOLD variance in an event‐related scene categorisation task (albeit using alternative methods to those used here). More complex methods have been developed in recent years in order to control for these confounds—such a calibrated BOLD (Blockley, Griffeth, Simon, & Buxton, 2013; Hoge et al., 1999) which provides a more complete quantification of the BOLD signal—but some of these approaches often involve an increased methodological burden such as requiring a CO_2_ hypercapnic challenge during acquisition. In human imaging studies of drug effects, where the burden on the participant is already considerable due to a necessarily long study day involving multiple assessments, there is clearly a need for pragmatic and noninvasive assessment. This is particularly relevant for patient studies (Lajoie et al., 2017). Here, we have illustrated a strategy to efficiently account for two of the major physiological confounds without requiring additional equipment, expertise or participant imposition.

### 4.2 Reward anticipation

The suppression of reward anticipatory signals in striatal areas by risperidone replicates the earlier findings with olanzapine in a group of eight volunteers (Abler et al., 2007), but extends this to reveal a dose response relationship that persists with the addition of covariates for CVR and CBF. One explanation for the reduction in the striatal signal during reward anticipation following risperidone administration is that D2 blockade on striatal postsynaptic membranes results in suppression of the postsynaptic potential and the associated BOLD signal (Menon et al., 2007; Schott et al., 2008).

This reduction in reward anticipatory striatal BOLD is similar to that seen in unmedicated schizophrenia patients performing the MID (Esslinger et al., 2012; Juckel, Schlagenhauf, Koslowski, Wustenberg, et al., 2006b; Nielsen et al., 2012). One proposed mechanism for the reduced striatal reward activation in schizophrenia is that the increased baseline dopamine tone in the striatum in patients (Abi‐Dargham et al., 2000; Fusar‐Poli & Meyer‐Lindenberg, 2013; Howes et al., 2012) means the phasic signals that mark rewarding stimuli or reward‐predicting cues are effectively attenuated and do not appreciably change the BOLD signal (Heinz & Schlagenhauf, 2010). Knutson et al. (2004) investigated this hypothesis in healthy volunteers by administering amphetamine, which promotes release of striatal dopamine, and subsequently found a reduced BOLD response to reward anticipating cues. In our healthy volunteers, the reduced striatal signal following risperidone may therefore be a result of *increased* DA tone due to increased midbrain DA neuron activity which acute antipsychotic dosing has been shown to promote (Bunney & Grace, 1978; Chiodo & Bunney, 1983; di Giovanni, di Mascio, di Matteo, & Esposito, 1998), potentially via a ventral striatum‐ventral pallidum‐ventral tegmental area feedback pathway (Valenti & Grace, 2010). However, this explanation does not fully encompass the differences reported between typical and atypical antipsychotics which have been reported in patient groups (Schlagenhauf et al., 2008), and a direct comparison of first generation against second generation medication in healthy volunteers could further understanding of the underlying processes and different receptor systems involved here.

### 4.3 Reward outcome

We found a dose response increase during reward outcome after risperidone, with an increase in activation in the region of the amygdala on the higher dose. One interpretation here is that the separate processes of anticipation and consummation are being influenced directly and differentially by the drug, attenuating activity during anticipation and enhancing it during outcome. There are numerous other examples within the reward processing circuit of opposing effects of interventions during anticipation and receipt of reward. Separate systems for predictive, incentive and consummative signals of reward in rats have been identified and pharmacologically modulated (Smith, Berridge, & Aldridge, 2011). A similar divergence of activity has been reported in humans using fMRI (Dillon et al., 2008; Knutson et al., 2001; Rademacher et al., 2010) and EEG (Angus et al., 2017; Novak & Foti, 2015), while BOLD activity during the different phases of the MID task have been differentially modulated by stress induction in healthy volunteers (Kumar et al., 2014) in an inverse divergence to the results reported here.

However, perhaps the most parsimonious explanation for our observations is that the changes seen in the consummatory phase are a product of the effects of the drug on the anticipatory phase, and suppression of the DA signal during anticipation has then influenced activity during the outcome phase. This aligns with the classical model from Schultz, Dayan, and Montague (1997) whereby the response to unpredicted rewards “shifts” to the cue when its rewarding value has been learned. A drug‐induced suppression of the dopaminergic signal during the anticipatory phase may be related to reduced predictive value and result in a relative increase in signal during outcome.

The increases in activation during reward outcome observed in this study were localised to the left amygdala and not striatal areas where a response to a novel cue might be expected to be observed. However, the amygdala is well placed to modulate information processing within the reward network—it projects directly to the nucleus accumbens in the striatum and is directly innervated by midbrain dopamine neurons (Haber, 2011) and may play a role in assigning the emotional relevance or drive of an environmental stimuli (Belova, Paton, Morrison & Salzman, 2007). fMRI work in humans with the MID and similar paradigms have indicated amygdala activation is more involved with reward receipt than anticipation (Ernst et al., 2005; Knutson & Greer, 2008). If suppression of the signal at cue‐presentation resulted in increased activity due to the perceived novelty of the reward, it is plausible the amygdala may be recruited to assess the nature of the outcome. Alternatively, the dampening of dopaminergic neurons by the drug may result in the partial release of amygdala from dopaminergic control, resulting in the increased sensitivity of amygdala neuronal populations resulting in a hyper‐reactive state. In either event a clear explanation as to why the amygdala would be selectively affected in this way remains incomplete.

## 5 CONCLUSIONS

We have shown for the first time that risperidone has both a dose and “reward phase” dependant effect in healthy humans. A reduction in reward anticipatory activation was present without significant changes in sedation or behavioural performance and preceded localised increases in activity during reward receipt, and these changes survived after careful control for drug‐induced changes in regional blood flow and changes in BOLD response to a breath hold task (as a proxy of vascular reactivity). This strategy substantially accounts for two of the most important nonneuronal effects of the drug and provides more directly interpretable results in a relatively simple fashion. The acute and chronic effects of the administration of other dopamine D2 antagonists with varying receptor profiles needs to be studied in placebo‐controlled designs to appreciate the potential implications for patients.

## CONFLICT OF INTEREST

The authors declare no conflict of interest.

## ETHICS STATEMENT

The study was approved by the London (Brent) Human Research Ethics Committee (REC reference: 13/LO/1183).

## Supporting information


**DATA S1**: Supporting information

## Data Availability

Data sharing is not applicable to this article as no new data were created or analyzed in this study.
