# Supplementary material for: The effect of risperidone on reward‐related brain activity is robust to drug‐induced vascular changes
Source: Hum Brain Mapp. 2021 Mar 5;42(9):2766–77. doi: 10.1002/hbm.25400 (PMC8127149; doi:10.1002/hbm.25400)
Supplement: Supplementary file 1 — DATA S1: Supporting information [file HBM-42-2766-s001.doc]

# Supplementary information

# Methods

## Participant screening

An initial session assessed general physical suitability for the study, including medical history, a full physical exam, vital signs, electrocardiogram, blood and urine chemistry profiles, serology (HIV1, HIV2, hepatitis B and/or hepatitis C), tests for alcohol and drugs of abuse, and concomitant medication. Inclusion criteria required normal ECG, standard laboratory blood screens and urinalysis, and alcohol consumption within the recommended guidelines at the time of the study (less than 21 units per week). Exclusion criteria included smoking more than 10 cigarettes per day, a history of neurological or psychiatric illness, physical illness and positive drugs of abuse or alcohol breath test on the screening or study days.

A second screening session within one week of the planned first visit was conducted to familiarise the participant with the scanning protocol and fully assess their psychiatric history via an unstructured interview with a study psychiatrist. MRI safety screening was conducted, and participants were prepared for the scanning environment by means of a mock scanning session. This also permitted us to assess their ability to perform the tasks, for which standardised instructions were given.

## Sedation assessment

As a measure of subjective sedation, participants rated their own alertness using a Visual Analogue Scale (VAS), both prior to dosing and at regular points throughout the visit including prior to each scan. The VAS based questionnaire is a 16-item self-rated dimensional analogue scale, which can be categorised into two main factors that represent subjective alertness and tranquillity (Herbert, Johns, & Dore, 1976). It has been well-validated to quantify sedative drug effects in healthy volunteers including dopaminergic agents and antipsychotics (Liem-Moolenaar et al., 2010). The alertness factor was the main outcome measure for assessing sedation. Within visit changes in alertness scores from arrival at testing centre to the closest measure to the T-max of the drug was used as a sedation metric per visit and entered into a repeated measures ANOVA, with dose as within-subject factor.

## Image acquisition and preprocessing

### MID & Breath-hold

All scans were conducted on a GE MR750 3-Tesla scanner using a 12-channel receive-only head coil. Functional scans (MID and breath-hold) were carried out using a temporal series of Gradient-Recalled Echo Planar Imaging (GE-EPI) whole brain scans, each comprising of 38 near-axial slices, with an isotropic spatial resolution of 3.3mm (TR = 2000 ms; TE = 28 ms; flip angle = 75 degrees; number of volumes = 414 (MID), 146 (breath-hold); FoV = 214 mm). The initial four volumes of each time series were discarded to minimise non-steady-state effects on the signal amplitude. A T1-weighed MPRAGE scan, for use in spatial normalisation of the functional data, (FOV = 270mm, TR/TE/TI = 7.312/3.016/400ms, 256x256x156 matrix, slice thickness = 1.2mm) was acquired on the second visit.

The origin of the functional and structural images were reset to the anterior commissure-posterior commissure line. Functional images were slice time corrected (reference slice: 19). An initial between session alignment was performed for each participant where the first volume from sessions two and three were aligned to the first volume of the first session prior to two-pass realignment within each session. All volumes were then realigned to the mean image of all three sessions. The T1-weighted image for each subject was then coregistered to the resampled mean functional image from the realignment step, using the normalised mutual information objective function in SPM, and a DARTEL (Diffeomorphic anatomical registration through exponentiated lie algebra (Ashburner, 2007)) template created from the T1-w images. The realigned and coregistered functional volumes were resliced to original voxel sizes and the DARTEL flow fields applied to warp the data into MNI space. Normalised images were smoothed using an 8mm FWHM kernel.

Motion and framewise displacement parameters estimated during the realignment process were added as regressors in the first-level design matrix (Siegel et al., 2014). Any volumes with displacement of 1mm or more were flagged and marked with a 3-TR regressor (to include the volumes either side) in the first level design matrix (Power, Barnes, Snyder, Schlaggar, & Petersen, 2012). Any scan that required more than 10% of the volumes of the full run being regressed out in this manner resulted in that participant being removed from the analysis. The realignment parameters were visually inspected and any time-series for which the maximum detected translation from the first volume was greater than the dimensions of one voxel, or those which indicated stimulus correlated movement, were flagged for exclusion. Three participants were excluded based on these criteria. These participants were identified and excluded parallel to data collection, allowing them to be replaced by new recruits to maintain a suitably powered study. Participants excluded due to head motion do not form part of the sample described above.

### ASL

Images were acquired using a pseudo-continuous Arterial Spin Labelling sequence (pCASL) with a multi-shot, segmented 3D stack of axial spirals (8-arms) readout with a resultant spatial resolution of 2x2x3mm. Three control-label pairs were used to derive a perfusion weighted difference image. The labelling RF pulse had duration of 1.5s and a post-labelling delay of 1.5s was also used. The sequence included four background suppression RF pulses for optimum reduction of the static tissue signal. A Proton Density (PD) image was acquired in 48sec using the same acquisition parameters to compute the CBF map in standard physiological units (ml blood/100gm tissue/min). Two runs were acquired per visit and averaged following preprocessing.

A T2-weighted image (FOV = 240mm, TR/TE = 4380/46.992ms, 320x256x156 matrix, slice thickness = 2mm) collected on the first visit was co-registered to the T1-weigted image prior to creation of the DARTEL template, and each session’s PD image was then co-registered to the T2-weighted image. The parameters for this transformation were then applied to the CBF maps, and the DARTEL flow field applied to normalise to CBF maps to MNI space.

## Task paradigms and first level modelling

### Breath-Hold

The breath-hold paradigm has been used extensively in fMRI research as a measure of the ability of cerebral vasculature to modulate blood flow in response to vasoactive stimuli or cerebrovascular reactivity (Birn, Smith, Jones, & Bandettini, 2008; Thomason & Glover, 2008; Urback, MacIntosh, & Goldstein, 2017). It has been shown to be comparable to alternative methods of assessing vascular reactivity, such as CO2 inspiration (Kastrup, Kruger, Neumann-Haefelin, & Moseley, 2001; Tancredi & Hoge, 2013) or correction methods based on resting state physiological fluctuation amplitude (Kannurpatti & Biswal, 2008; Lipp, Murphy, Caseras, & Wise, 2015). The breath-hold challenge has the benefit of being non-invasive and easy to implement, with minimal discomfort or distress for the participant. Participants were instructed to follow a simple set of instructions on screen alternating between paced breathing (45 seconds) and breath holding (16 seconds), with this cycle repeated five times (data from the first cycle was discarded to eliminate non-steady state effects of the paradigm on the BOLD signal). Breath holding was instructed to commence at the end of expiration (or on a ‘out’ breath) as end expiration holding has been shown to produce a faster peak signal than end inspiration (Kastrup, Kruger, Glover, Neumann-Haefelin, & Moseley, 1999) and also leads to reduced motion induced artefacts*.* During the regular breathing portions of the task, participants were given instructions to breath at a controlled rate (breath in for 3 seconds, out for 3 seconds) as this approach produces a larger and more consistent peak BOLD signal and improved SNR than self-paced breathing (Scouten & Schwarzbauer, 2008). As the breath hold signal has been shown to plateau after approximately 20 seconds (H. L. Liu, Huang, Wu, & Hsu, 2002; Magon et al., 2009), a 16 second hold was chosen, to be long enough to produce a peak response whilst not being uncomfortable for the participant. Participants were supplied with a respiratory bellows sensor during each scan, which allowed for the monitoring of their breathing in the control room to ensure they were executing the task correctly, as well as providing information for analysis of group adherence to the task.

The breath hold challenge was modelled with box car function regressors for paced and held breathing, but incorporated a delayed onset of 9 seconds and included the temporal derivatives, as previously shown to provide the most accurate modelling of vascular reactivity (Murphy, Harris, & Wise, 2011). Whole brain maps of the contrast held > paced breathing provided a metric of CVR.

### MID Task

The Monetary Incentive Delay (MID) task (Brian Knutson, Westdorp, Kaiser, & Hommer, 2000) has been extensively used to elicit and study reward related activation within fMRI designs (B. Knutson & Greer, 2008), and has been shown to be reliable over time in healthy volunteers (Plichta et al., 2012). The version used in this study is most closely comparable to that used in B. Knutson, Fong, Adams, Varner, and Hommer (2001) and has been described extensively elsewhere (B. Knutson, Fong, Bennett, Adams, and Hommer (2003)).


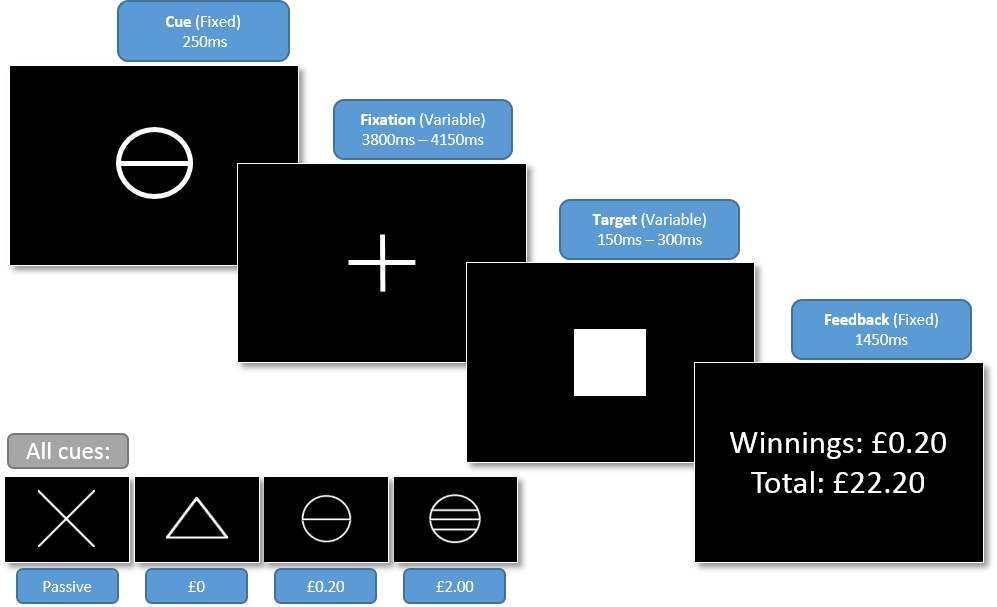


Figure s1: MID Task

The version employed here comprises of four randomised trial types: three active reward-level cues (high (£2), low (£0.20) and control (£0)), and a passive trial which required no response. If the participant pressed the button during the presentation of the fixation cross or within 100ms of the presentation of the target (an unrealistic reaction time), the trial would set as a no win. Each condition was presented 24 times within a total duration of approximately 13.8 minutes. The three active trial types were conducted within a fixed 10 second window, while the passive trial was a simple ‘X’ displayed for 4.25 secs without the fixation, target or feedback screens displayed in the active trials. Four separate ‘playlists’ with the 96 trials randomly arranged in each were created which participants randomly received on each visit according to a Latin square design, to ensure there were no learning effects from completing the same task on each visit.

The MID was modelled as outlined in Abler, Erk, and Walter (2007). Three cue regressors (high win, low win and neutral ) were defined for the anticipatory period depending on the cue presented (as a result of temporal jittering, this period had a variable total amount of time between 4050ms and 4400ms). The target was defined by a single regressor of 500ms. Five feedback regressors (high win, low win, high no win, low no win, neutral feedback) were defined for the feedback period depending on the cue type and outcome (win or no win) and set for a fixed period (1450ms). The entire duration of passive trials were defined as a single event of 4250ms. Motion and framewise displacement parameters estimated during the realignment process were also added resulting in the model consisting of the ten task regressors above and seven movement-related parameters. Performance related criteria were also set to ensure only data from participants who were actively and appropriately engaged in the task was included in the final analysis.

Contrasts of interest were set to explore main effect of anticipation of reward (High Cue & Low Cue > Neutral Cue), and main effect of receipt of reward (High Win & Low Win > High no-win & Low nowin). Levels of reward were combined to ensure a sufficient number of trials for a suitably powered analysis.

MID task performance thresholds

Performance related criteria were also set to ensure only data from participants who were actively and appropriately engaged in the task was included in the final analysis. When participants failed to make a response to an active task (ie no button press was attempted on non-passive trials), the trial was defined as a ‘missed response’ regressor of no interest. Any trials with an RT of +/-3SDs of that individual's mean were also classified as a missed response. This was to ensure only trials the individual was actively attending to were included in analysis. If more than 10% of a single active trial type was regressed out after applying this threshold, that subject was removed from the analysis.

Performance from the cohort as a group was also monitored and any participant performing more than 2 SDs of the session average either in percentage of hits on win trials or average reaction time (RT) response across all response trials were removed. An attempted response rate (i.e. a button press within the entire 500ms response window, regardless of win or lose) of >66% was also required for a participant to be included in the final analysis to ensure only those individuals that were actively involved in the task would be included in the final analysis.

Four participants were removed on the basis of these performance thresholds – these participants performed particularly badly, all achieving a win rate of no more than 15%.

## Second Level Analysis

Recent evidence suggests traditional cluster level statistics in whole brain analysis may be at risk of inflating the false positive rate (Eklund, Nichols, & Knutsson, 2016). To prevent this, we employed non-parametric permutation testing to explore whole-brain drug effects, which does not rely on any assumptions of normality. Paired sample t-tests of high dose vs placebo, and low dose vs placebo were conducted using the *RANDOMISE* feature in FSL with threshold free cluster enhancement (TFCE (Smith & Nichols, 2009)). Randomise also allows the inclusion of voxelwise covariates – therefore to assess the effect of the vascular covariates, a second round of analyses were conducted which included both the CVR and CBF maps as voxelwise covariates of no interest to explore the influence of the drug induced changes to baseline CBF and CVR on the BOLD signal. CBF maps were resampled to the same voxel size as the MID/breath-hold acquisitions as part of the DARTEL normalisation procedure.

5,000 permutations were conducted for each treatment-placebo comparison in order to create a non-parametric null distribution and calculate a 5% significance threshold, familywise error corrected. Exchangeability blocks were specified to ensure permutations would only occur within subject, to take account of the repeated measures nature of the data.

ROI definition

Seven reward system related bilateral ROIs were defined for further exploration of drug effect. The mean beta estimates created from first-level modelling were extracted from each of the ROIs using the MarsBar plugin in SPM12 and were used to assess drug induced changes in MID activity, and in the CVR and CBF metrics.

The main area of interest was the striatum, which has an established role in reward processing, and ROIs were based on the striatal structures and the dopaminergic input to the ventral striatum. Probabilistic bilateral putamen and caudate ROIs were defined from the FSL Harvard-Oxford subcortical atlas, thresholded at a probability index > 0.20 and then binarised. A bilateral ventral striatum ROI was also manually defined in MNI space as described in Montgomery, Mehta, and Grasby (2006), based on previous work by Mawlawi et al. (2001) – briefly, the boundary between the ventral striatum, dorsal caudate, and dorsal putamen was defined by a line joining the intersection between the outer edge of the putamen with a vertical line going through the most superior and lateral point of the internal capsule and the centre of the portion of the AC trans-axial plane overlying the striatum. This line was extended to the internal edge of the caudate. The other boundaries of the ventral striatum were visually determined by its dense grey signal and were easily distinguishable from the adjacent structures. The ventral striatum was sampled from the anterior boundary of the striatum to the level of the AC coronal plane.

The ventral tegmental area (VTA) in the midbrain is the major source of DA neurons projecting to the ventral striatum. The ROI was taken from the probabilistic midbrain atlas developed and validated in Murty et al. (2014) and is publicly available at <https://www.adcocklab.org/neuroimaging-tools>. As described in Murty et al. (2014), the VTA was hand drawn on the T1-weighted structural scan of a sample 50 individual healthy volunteers, with the defined anterior boundary at the CSF, posterior boundary at the coronal section that bisected the red nucleus, superior boundary at the top of the superior colliculus, inferior boundary at the bottom of the red nucleus, and lateral boundaries in the sagittal slice connecting the peak of curvature of the interpeduncular fossa with the centre of the colliculus. Following normalisation of each ROI into MNI space, averages of the 50 binary ROIs were calculated to create a probabilistic VTA ROI. A thresholded ROI at a probability index > 0.20 was used in this study.

The amygdala is a critical part of the limbic system sharing direct connections with the ventral striatum and VTA, has been implicated in the non-receipt of expected reward, as well as ‘positive’ reward processing, and has been shown to be sensitive to DA modulation during reward processing in heathy volunteers (Murray, 2007; O’Daly et al., 2014; Russo & Nestler, 2013; Tye, Cone, Schairer, & Janak, 2010). A bilateral amygdala ROIs was defined from the FSL Harvard-Oxford subcortical atlas, thresholded at probability index > 0.20 and binarised.

The vmPFC shows strong connections to the ventral striatum (Pujara, Philippi, Motzkin, Baskaya, & Koenigs, 2016), with the anterior cingulate being more connected to dorsal striatal regions (Haber & Knutson, 2010). Both regions have been shown to be involved in the processing of outcome phase of reward (Vassena, Krebs, Silvetti, Fias, & Verguts, 2014) and were only included in the analysis exploring activation during this stage of the MID. The ACC ROI was defined from the AAL atlas (Tzourio-Mazoyer et al., 2002)**.** vmPFC ROI was defined from coordinates in (Vassena et al., 2014) based on X. Liu, Hairston, Schrier, and Fan (2011) and centred a 10mm sphere on MNI coordinates x=0 y=51 z=−10.

# Results

## MID Task performance

The 17 participants in the final analysis maintained engagement throughout the duration of the task across all sessions, responding to at least 95% of trials that required a button press - importantly this includes both win and neutral trials indicating attention and performance was maintained on trials when no monetary reward was available (see figure s2). A one-way repeated measures ANOVA revealed a significant effect of treatment drug on response rate (*F*(2,32) = 4.480, p = 0.043), although this difference was not significant after Bonferroni correction for the number of pairwise comparisons (the largest change between high risperidone (95.51 ± 1.31%) and placebo (98.2 ± 0.35%), a non-significant change of 2.96% (p = 0.176)). There was no significant effect of drug on hit rate (*F*(2,32) = 2.048, p = 0.146) indicating the task paradigm maintained participants’ performance to close to 60% across all three sessions.


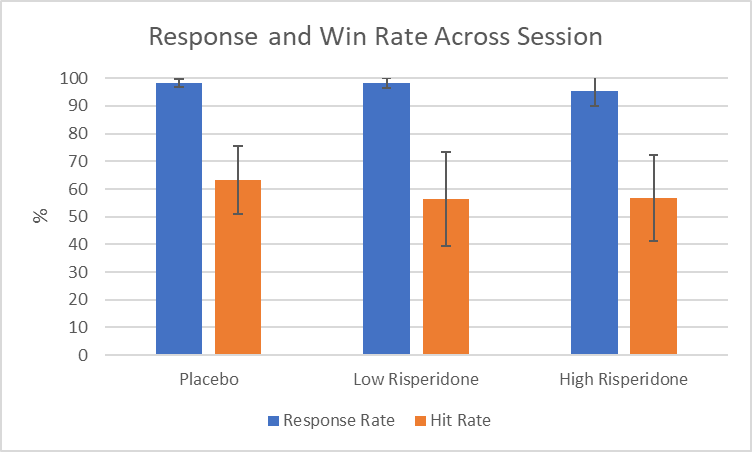


Figure s2: MID behavioural performance in response rate and hit rate (SD) across levels of risperidone (n=17).

## Breath hold

Participants adhered to the task well, and the timing and extent of breath holding was consistent across drug conditions (see figure s3). Paired t-tests of placebo vs high risperidone sessions and placebo vs low risperidone sessions for the bellows reading at the commencement of the task (i.e. the extent to which the participant had breathed in at the display of the command instructing them to breath out and hold) revealed no significant difference between either set of conditions and placebo (Figure s3, High dose (*t*(20) = -0.23, *p* = 0.82) and Low dose (*t*(20) = -0.42, *p* = 0.68)).

**
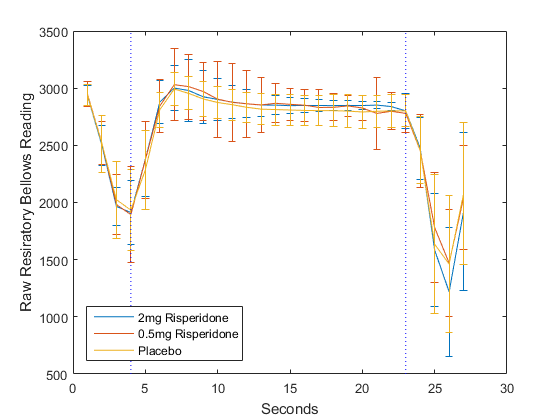
**

Figure s3: Average respiratory bellows readings (and SD) of the 4 breath hold blocks per condition for each of the three sessions (n=21).r the RisH/L group. Vertical Blue dotted lines denote start and end of task as displayed to participant.

### Main effect of Breath-Hold task

Fig s4: Whole brain permutation testing of Placebo scans during breath hold task (Hold>Paced breathing), 5000 permutations, FWE corrected p<0.05 (n=17). Colour bar denotes voxelwise one sample t-statistic.

### CBF maps


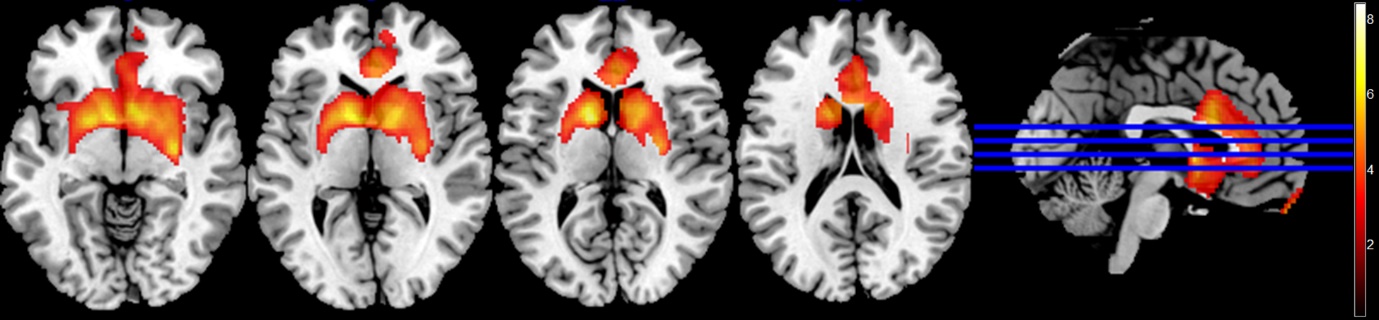


Fig s5: Effect of 2mg Risperidone > Placebo on CBF. Whole brain permutation testing, (5000 permutations, FWE corrected p<0.05, n=21) Colour bar denotes voxelwise paired sample t-statistic.

*
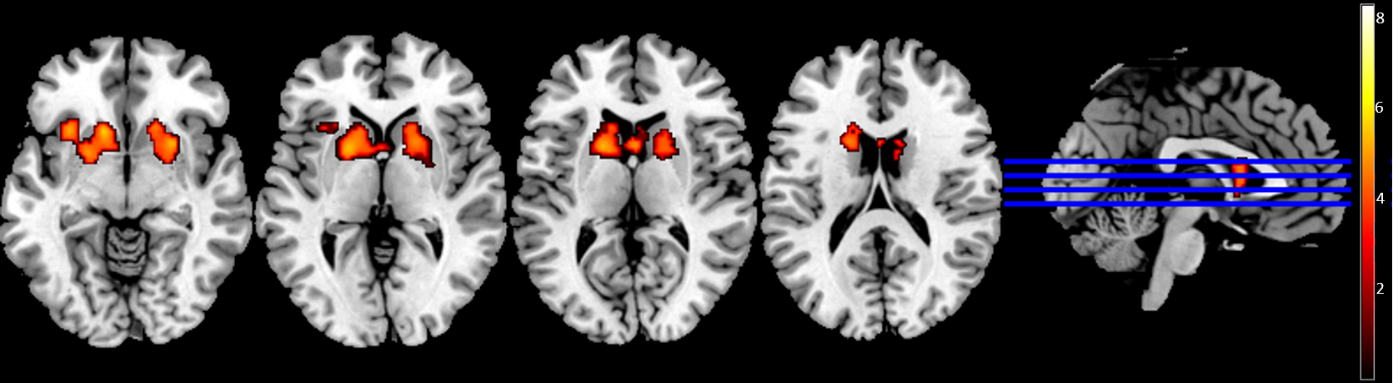
*

Fig s6: Effect of 0.5mg Risperidone > Placebo on CBF. Whole brain permutation testing, (5000 permutations, FWE corrected p<0.05, n=21) Colour bar denotes voxelwise paired sample t-statistic

Unthresholded maps of all voxelwise analyses from this study will be made available on Neurovault.org

## Peak voxel coordinates (MID risperidone analysis)

| **Reward anticipation: Placebo>2mg risperidone** | Cluster size (vox) | Peak t-stat | MNI coordinates (mm) | | |
| --- | --- | --- | --- | --- | --- |
|  |  |  | X | Y | Z |
|  | **7250** | **5.99** | **-23** | **3** | **46** |
|  |  | 5.85 | -17 | -20 | 23 |
|  |  | 5.73 | 20 | -13 | 23 |
|  |  | 5.34 | 27 | -60 | 50 |
|  |  | 5.33 | 10 | -77 | -13 |
|  |  | 5.16 | 20 | 17 | -3 |
|  | **15** | **3.37** | **-47** | **-33** | **3** |
| **Reward anticipation: Placebo>2mg risperidone, including voxelwise CBF and CVR maps** | Cluster size (vox) | Peak t-stat | MNI coordinates (mm) | | |
|  |  |  | X | Y | Z |
|  | **667** | **5.63** | **-23** | **-86** | **10** |
|  |  | 5.62 | 3 | -93 | -3 |
|  |  | 5.38 | -24 | -84 | 20 |
|  |  | 5.37 | 0 | -90 | -13 |
|  |  | 5.33 | 7 | -78 | -13 |
|  | **650** | **6.85** | **-20** | **-17** | **76** |
|  |  | 6.62 | -27 | -20 | 73 |
|  |  | 6.05 | 13 | -33 | 73 |
|  |  | 5.84 | 0 | 7 | 50 |
|  |  | 5.71 | 7 | -10 | 76 |
|  |  | 5.6 | -33 | -20 | 69 |
|  | **159** | **6.56** | **17** | **-10** | **23** |
|  |  | 5.23 | 17 | 0 | 26 |
|  |  | 4.76 | 3 | -30 | 0 |
|  |  | 4.48 | 3 | -3 | 3 |
|  |  | 4.45 | -7 | -37 | 3 |
|  |  | 4.42 | 3 | -20 | 13 |
|  | **147** | **5.03** | **30** | **-57** | **66** |
|  |  | 5.02 | 13 | -73 | 59 |
|  |  | 4.89 | 27 | -60 | 53 |
|  |  | 4.53 | 30 | -67 | 53 |
|  | **41** | **4.43** | **-50** | **-50** | **-30** |
|  |  | 4.09 | -53 | -57 | -30 |
|  |  | 4.03 | -47 | -40 | -30 |
|  |  | 3.86 | -50 | -67 | -26 |
|  | **23** | **4.91** | **30** | **-7** | **-26** |
|  | **17** | **5.89** | **-17** | **-23** | **20** |
|  | **13** | **4.85** | **40** | **-47** | **20** |
|  | **5** | **3.95** | **53** | **-17** | **26** |
|  | **2** | **5.21** | **-20** | **20** | **7** |
|  | **1** | **3.66** | **-57** | **-57** | **-17** |
|  | **1** | **6.36** | **-23** | **3** | **46** |
| **Reward outcome: Placebo<2mg risperidone** | Cluster size (vox) | Peak t-stat | MNI coordinates (mm) | | |
|  |  |  | X | Y | Z |
|  | **72** | **5.84** | **30** | **-7** | **-20** |
|  | **3** | **5.56** | **53** | **-17** | **-23** |
| **Reward outcome: Placebo<2mg risperidone, including voxelwise CBF and CVR maps** | Cluster size (vox) | Peak t-stat | MNI coordinates (mm) | | |
|  |  |  | X | Y | Z |
|  | **3** | **5.83** | **27** | **-3** | **-17** |

# References
